# A Review of Microsphere Super-Resolution Imaging Techniques

**DOI:** 10.3390/s24082511

**Published:** 2024-04-14

**Authors:** Wenbo Jiang, Jingchun Wang, Yidi Yang, Yun Bu

**Affiliations:** 1 School of Electrical Engineering and Electronic Information, Xihua University, Chengdu 610039, China; wangjc@stu.xhu.edu.cn (J.W.); yangyd@stu.xhu.edu.cn (Y.Y.); buyun@mail.xhu.edu.cn (Y.B.); 2 Sichuan Provincial Key Laboratory of Signal and Information Processing, Xihua University, Chengdu 610039, China

**Keywords:** microsphere super-resolution imaging technology, imaging principles and modalities, microsphere preparation process, microsphere manipulation, application areas

## Abstract

Conventional optical microscopes are only able to resolve objects down to a size of approximately 200 nm due to optical diffraction limits. The rapid development of nanotechnology has increased the demand for greater imaging resolution, with a need to break through those diffraction limits. Among super-resolution techniques, microsphere imaging has emerged as a strong contender, offering low cost, simple operation, and high resolution, especially in the fields of nanodevices, biomedicine, and semiconductors. However, this technology is still in its infancy, with an inadequate understanding of the underlying principles and the technology’s limited field of view. This paper comprehensively summarizes the status of current research, the advantages and disadvantages of the basic principles and methods of microsphere imaging, the materials and preparation processes, microsphere manipulation methods, and applications. The paper also summarizes future development trends.

## 1. Introduction

**Figure 1:**
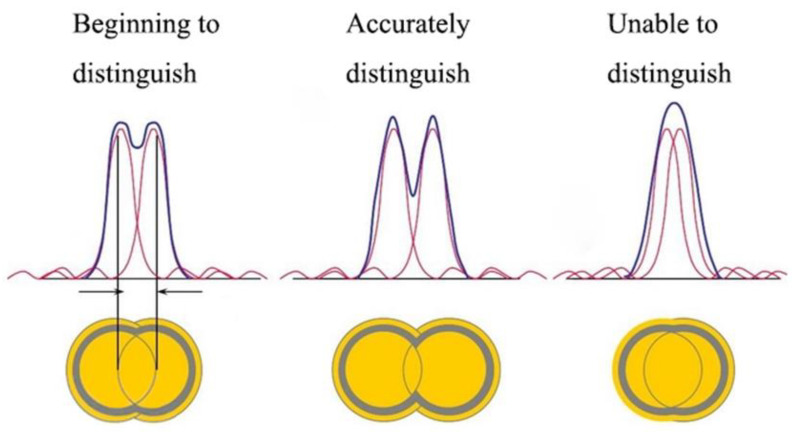
Rayleigh criterion for optical diffraction resolution [2].

Optical microscopy has long been an important tool for studying the miniature world. However, the resolution of conventional optical microscopes is limited by the optical diffraction limit. According to Ernst Abbe’s theory of resolution for coherent illumination, the resolution of conventional microscopes is limited by the diffraction of light and the numerical aperture of the lens [1]. From this theory, Rayleigh proposed his famous “Rayleigh criterion” [2] that measures the ability of an optical system to separate the image of two neighboring object points, as shown in Figure 1.

The equation for the limiting resolution of an optical imaging system is expressed as:

(1) R=0.61λ/NA

where *R* is the resolving distance of the objective lens, *λ* is the illumination wavelength, and *NA* is the numerical aperture of the objective lens. The product of the refractive index n of the medium between the objective lens and the sample and the sine of the half angle of light collection is as follows:

(2) NA=nsinθ

These equations show that reducing the incident wavelength or increasing the numerical aperture enables the microscope to achieve a higher resolution. However, microscopes illuminated with visual light employ illumination wavelengths of between 400 and 700 nm and numerical apertures of the non-immersion lens are around 0.95 at maximum. These wavelengths and apertures make it impossible for conventional optical microscopes to image structures with feature sizes smaller than 200 nm. To meet the demand for observing and manipulating even smaller structures, super-resolution imaging presents an opportunity to break through the diffraction limit of existing optical path systems.

Super-resolution imaging technology is the most significant breakthrough in the field of optical microscopic imaging in this century. The current mainstream optical super-resolution imaging technologies include STED, SIM, and SMLM [3]. These technologies exhibit advantages and disadvantages in terms of imaging speed, sample processing requirements, and applicable fields [4]. A comparison between microsphere super-resolution imaging technology and these methods is shown in Table 1.

Although there are some differences in the principles, applications, and imaging methods of these super-resolution imaging technologies, they can all achieve high imaging resolution. At the same time, these technologies can complement each other, allowing for the selection of suitable technologies for specific application scenarios. Among the super-resolution imaging techniques shown in Table 1, combining microspheres with traditional optical microscopes has several advantages, including a low cost, simple operation, high resolution, and applicability across multiple fields. Furthermore, this combination can still, to some extent, overcome the limitations faced by traditional microscopes, although it may still encounter some challenges. For example, in terms of setting limitations, although microspheres can help improve imaging resolution, the optical design of traditional microscopes may still be insufficient to fully utilize the super-resolution effect of microspheres. Therefore, a specially designed optical system is needed to maximize the utilization of microsphere characteristics. In terms of sample limitations, the preparation of microsphere-labeled samples may be complex, and the uniform distribution and correct positioning of the microspheres must be ensured in order to avoid confusion and distortion during the imaging process. In terms of wavefront aberration limitation, microspheres may be affected by scattering and absorption, which may distort light and reduce imaging quality. In terms of manufacturing and cost constraints, different types of samples may require specific types or sizes of microspheres, necessitating additional effort and resources. Addressing these limitations will also be a focus of future research. This paper provides a detailed introduction to microsphere super-resolution imaging technology, including the principles and methods, materials and preparation, manipulation methods, and identification of potential uses of microspheres now and in the future.

## 2. Microsphere Super-Resolution Imaging Principle

### 2.1. Theoretical Analysis of Microsphere Super-Resolution Imaging

**Figure 2:**
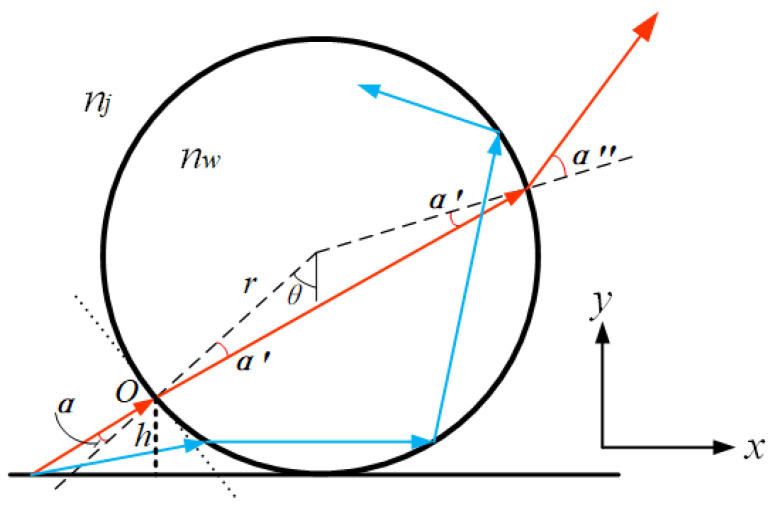
Diagram of a microsphere-coupled evanescent wave, where the red and blue solid lines represent the optical paths when the light is incident on the microsphere at different incident angles, respectively.

Conventional optical microscopes are unable to effectively image structures with feature sizes smaller than 200 nm due to the diffraction limit, with the loss of signals carrying high-frequency information about the object thought to be the reason for the diffraction limit. Evanescent waves carry more high-frequency information, existing only on the medium demarcation surface and propagating with a wave number greater than that of a conduction wave of the same frequency. Due to its rapid intensity decay in the direction perpendicular to the surface, it cannot be detected by conventional optical microscopes. When using microspheres to observe the surface of the sample, Maxwell’s equations show that the tangential component of the electric field is continuous at the boundary. Part of the evanescent wave carries the precise structural information about the object and is coupled into the microsphere through the boundary of the microsphere, which is transformed into a propagating wave that can be transmitted in the far field, thereby enabling super-resolution imaging. The principle of microsphere-coupled swift waves is shown in Figure 2.

As shown in Figure 2, for an evanescent wave scattered by the sample reaching point O on the surface of the microsphere, the longitudinal height from the outer surface of the microsphere is *h*, the refractive index of the microsphere is *n_w_*, the refractive index of the ambient submerged medium holding the microsphere is *n_j_*, and the number of waves is *K*. The horizontal component of the evanescent wave is *x*, and the vertical component is *y*. The tangential component *x*_1_ of the electric field at the interface is determined by the electromagnetic field boundary conditions and geometrical relations:

(3) $$x_{1}=(x\cos\theta {)}^{2}-(|y|\sin\theta {)}^{2}$$

The evanescent wave can be coupled and propagated by the microsphere only when the tangential component of the electric field is less than the maximum wave number propagating inside the microsphere, as defined by the relation:

(4) $$(x\cos\theta {)}^{2}-(|y|\sin\theta {)}^{2}\leq {\left(n_{w}K\right)}^{2}$$

Conducted waves after microsphere coupling should avoid total reflection at the interface to propagate to the far field, so the tangential component of the electric field should be less than the maximum wave number of the submerged medium.

(5) $$(x\cos\theta {)}^{2}-(|y|\sin\theta {)}^{2}\leq {\left(n_{j}K\right)}^{2}$$

In summary, the tangential component of the electric field should be less than the maximum number of waves propagating in the microsphere versus the maximum number of waves in the submerged medium, as expressed by:

(6) cosθ=(r−h)/r

It should be noted that not all scattered light can be coupled with the microsphere. As shown in Figure 2, the light can be transmitted out of the microsphere only when the angle is less than α. Otherwise, the light will be totally reflected inside the microsphere. Furthermore, evanescent waves decay exponentially in the vertical direction, so experiments should avoid choosing a large height *h*. In addition, for larger values of the angle *θ*, the microsphere couples and propagates more evanescent wave information to the far field. Smaller values of the microsphere radius *r* also favor the coupling of evanescent waves.

### 2.2. Microsphere Super-Resolution Imaging Modalities

Current research into super-resolution imaging using combinations of microspheres with optical microscopy falls into the categories of microsphere-based imaging and immersed microsphere-based imaging.

#### 2.2.1. Microsphere-Based Imaging

**Figure 3:**
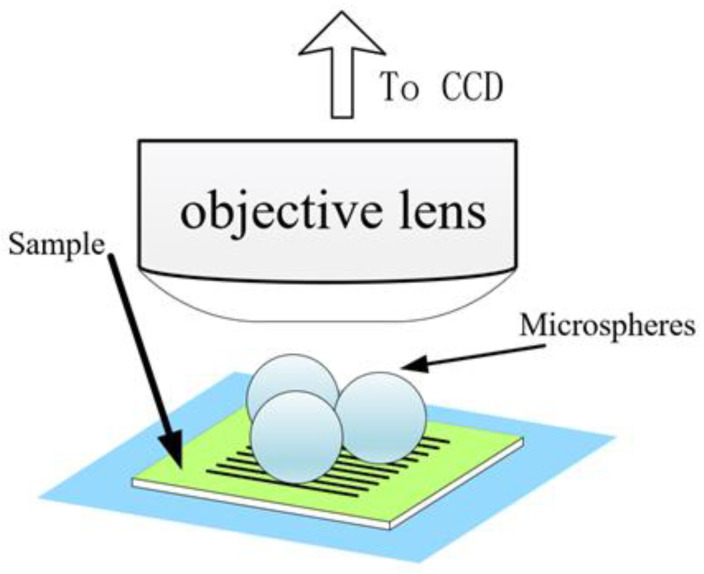
Schematic of ordinary microsphere imaging.

Microscopic imaging of samples by direct seeding of microspheres is a common imaging method. As the name suggests, the method involves sprinkling microsphere powder directly on top of the sample for simple and effective imaging, or dropping the microsphere solution directly on the sample and waiting for the solution to evaporate to observe the imaging results. A schematic of the direct seeding method is shown in Figure 3. It should be noted that directly sprinkling microspheres on the surface of the sample easily damages the surface of the sample and does not allow accurate placement of the microspheres. Precise control of microsphere placement is a major research concern.

**Figure 4:**
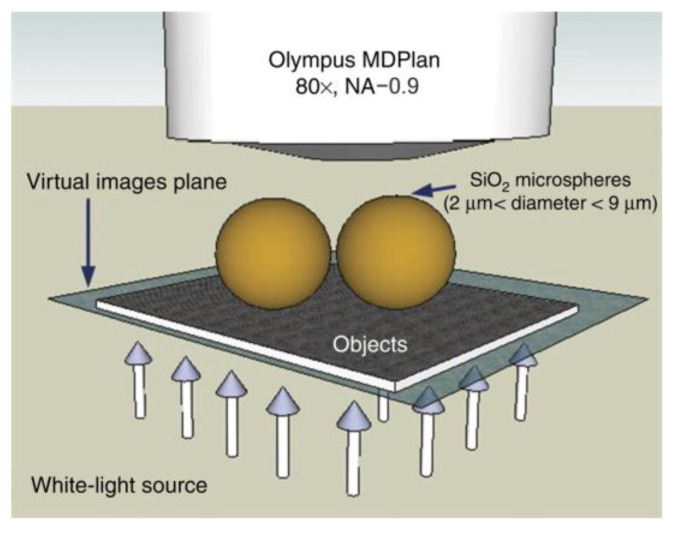
Experimental structure of white light microsphere nanoscopes with imaging resolution λ/8–λ/14 [6].

**Figure 5:**
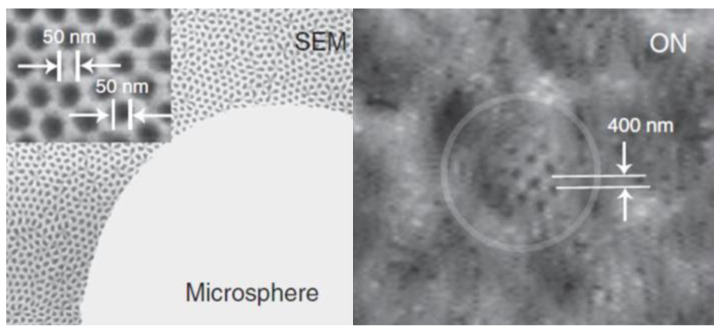
Images of 50 nm structures, where the white arrow indicate the sample size [6].

In 2009, Ju Young Lee et al. [5] successfully synthesized nanoscale spherical lenses that broke through the imaging resolution limit of conventional microscopes when used in combination with conventional optical microscopes. Subsequently, in 2010, Zengbo Wang et al. [6] from the United Kingdom placed silica microspheres with a refractive index of approximately 1.46, and diameters ranging from 2 μm to 9 μm, directly on the surface of the samples to be tested. They used white light illumination with a central wavelength of 600 nm, a numerical aperture of 0.9, and a magnification of 80× for the microscope objective. They succeeded in resolving a porous alumina structure with a diameter of 50 nm and a spacing of 50 nm, obtaining an imaging magnification of 8×. The structure of the imaging experiment is shown in Figure 4, and the imaging results are shown in Figure 5.

Ju Young Lee’s group called this simple and effective technique microsphere super-resolution imaging. This technique has attracted the attention of many researchers worldwide because of its ready availability, simplicity of operation, and high potential application value.

However, super-resolution imaging with microspheres is very limited and can only be used for super-resolution imaging of Blu-ray disc stripes and the like. In response, researchers continue to explore using the principles of super-resolution imaging mechanisms, seeking to expand the application potential of this technology in the future. For example, in 2013, Zengbo Wang and Lin Li et al. [7] obtained super-resolution images of polystyrene (PS) microspheres with diameters of 30 µm, 50 µm, and 100 µm and explored the role of microsphere photonic nanojets in super-resolution imaging using the Mie scattering theory [8]. In 2014, Lin Li and Minhui Hong et al. [9] resolved images close to 25 nm by using a combination of microspheres and scanning laser confocal microscopy. This study opened new opportunities for understanding and developing microsphere-coupled scanning laser confocal nano-imaging for higher-resolution imaging. In 2015, Lin Li et al. [10] used high refractive index microsphere optical nanotechnology to image nanostructures. They conducted a theoretical analysis of the imaging phenomenon using the characteristics of the electric field vector and photon nanojets. In 2016, Feifei Wang et al. [11] investigated the effect of illumination modes on the super-resolution of microspheres while implementing three-dimensional super-resolution detection with microsphere-assisted white light interferometry. They also proposed a non-invasive microsphere scanning superlens microscope with temporal efficiency. This technique involved replacing the imaging area of the atomic force microscope tip with a microsphere and stitching together the recorded regions during the scanning process, with an acquisition efficiency of approximately 200 times that of the atomic force microscope [12]. In the same year, Hok Sum Sam Lai et al. showed that true super-resolution images could be formed using high refractive index microspheres and investigated the relationship between focal point positions and additional magnification using both experimental and theoretical methods [13]. These research findings have provided valuable guidance in understanding these imaging mechanisms.

**Figure 6:**
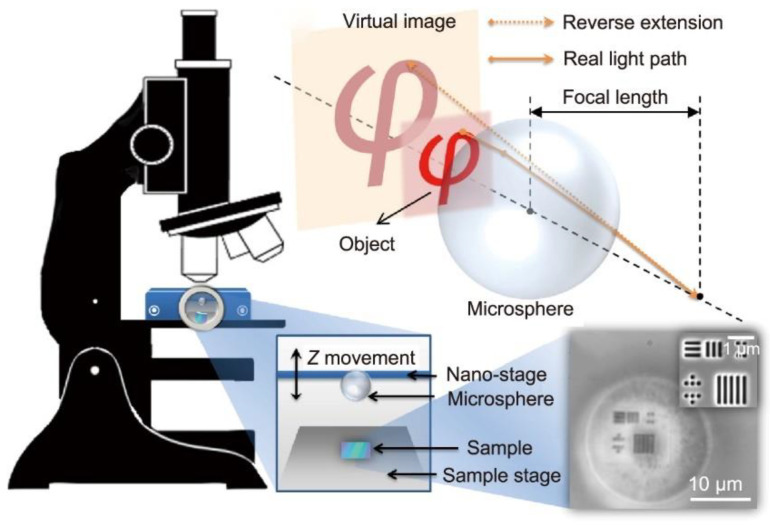
Microsphere nano-imaging platform [15].

The immaturity of this imaging technology makes commercial applications challenging. In addressing this issue, researchers have conducted numerous studies. In 2017, Zengbo Wang et al. [14] integrated traditional microscope objectives with microspheres to achieve non-contact super-resolution imaging of samples, marking a milestone in the practical application of microsphere super-resolution technology. In 2018, a research team led by Hung Ming hui from the National University of Singapore [15] used a stepper motor to control the position between the microspheres and the objective lens, integrating them into a whole. They developed a microsphere nano-imaging platform as shown in Figure 6 that is able to perform unlabeled scanning imaging on samples. They successfully resolved samples with a minimum feature size of 23 nm and attempted commercial development.

In 2020, Bing Yan et al. [16] integrated high-refractive-index BTG microspheres with a conventional microscope and set up a real-time lateral monitoring system to observe the distance between the microspheres and the sample to prevent damage to the sample. His team used the integrated objective to perform large-area imaging in the far field. The team referred to this integrated objective lens as the planar convex microsphere (PCM) objective. The PCM objective is readily integrated with existing microscope systems and holds significant commercial potential. In the same year, Lianwei Chen et al. [17] at the National University of Singapore explored the prospect of optical microsphere nanoscopy, a method that can dramatically improve the observational capabilities of conventional optical microscopes and has high theoretical applicability in various fields. This technology is in its early stages, and further development still requires much research, but it has great practical and commercial potential. In 2021, Chang Liu et al. [18] from Peking University conducted a study on multilayer structured samples using microsphere-assisted microscopy, proving that microsphere microscopes have scanning uses in tomography. In addition, their team also experimentally concluded that increasing the relative refractive index between the microspheres and the immersion medium increases the magnification but does not guarantee a higher resolution. In the same year, Qiaowen Lin et al. [19] at Datong University, Shanxi, carefully analyzed the role that photon nanojets, formed when microspheres focus light, play in microsphere super-resolution imaging. This nanojet converts evanescent waves into propagating waves for far-field imaging. The technology can be applied to the surface topography of micro-nanostructured objects, lithography, and label-free biomedical imaging (e.g., for bacteria and viruses).

As research on microsphere super-resolution imaging matures, it is believed that these methods can be commercialized and used in applications such as nanodevices, biomedicine, and semiconductors.

#### 2.2.2. Immersion Microsphere-Based Imaging

In comparison with microspheres exposed to air, experimental results have shown that images obtained using submerged microspheres have higher contrast and better resolving power. Liquid-immersed microsphere imaging methods refer to the dropping of alcohol or other liquids around the microspheres so that the microspheres are in a semi-immersed or fully immersed state. Generally speaking, the reason for adopting different immersion levels is that partially immersing low-refractive-index microspheres or fully immersing high-refractive-index microspheres in a medium is more conducive to high-resolution imaging [20].

**Figure 7:**
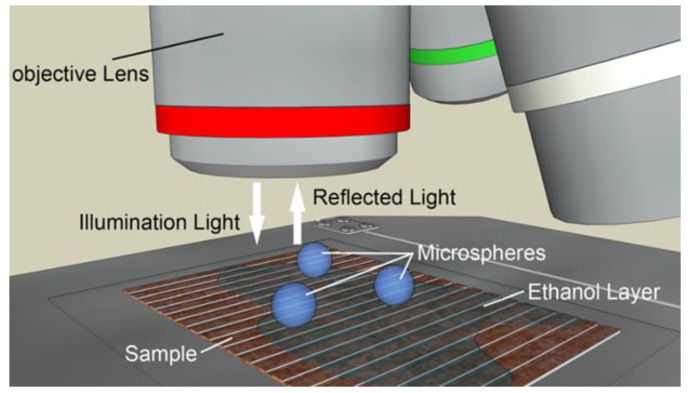
Structure of the microsphere semi-immersion experiment [21].

**Figure 8:**
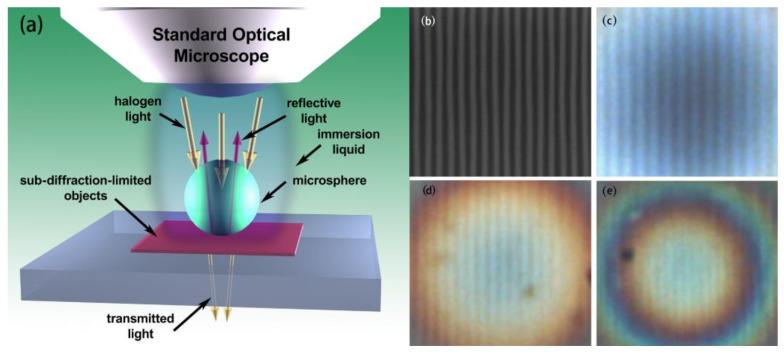
(**a**) Schematic of super-resolution imaging of microspheres under light illumination. (**b**) Scanning electron microscope (SEM) image of a blue light disc with a line width of 120 nm and a spacing of 180 nm. Enlarged images were obtained using barium titanate microspheres with a diameter of 100 μm in conjunction with an optical microscope in (**c**) water, (**d**) 40% sugar solution, and (**e**) microscope immersion oil [23].

In 2011, Hao Xiang et al. [21] from Zhejiang University semi-immersed microspheres in ethanol using the experimental structure shown in Figure 7. The experimental results showed that this operation could improve the imaging contrast. In 2012, Arash Darafsheh et al. [22] showed experimentally that barium titanate (BaTiO_3_) microspheres could achieve super-resolution imaging in an immersed state. In 2013, Seoungjun Lee et al. [23] experimented with BaTiO_3_ microspheres submerged in water, 40% sugar solution, and microscope immersion oil, with the experimental results showing the optical magnification to be 3.3×, 2.8×, and 2.3×, respectively. Diagrams of the experimental structures and the experimental results are shown in Figure 8. Experiments with submerged microspheres confirm the potential of microspheres to achieve super-resolution imaging in liquid environments.

**Figure 9:**
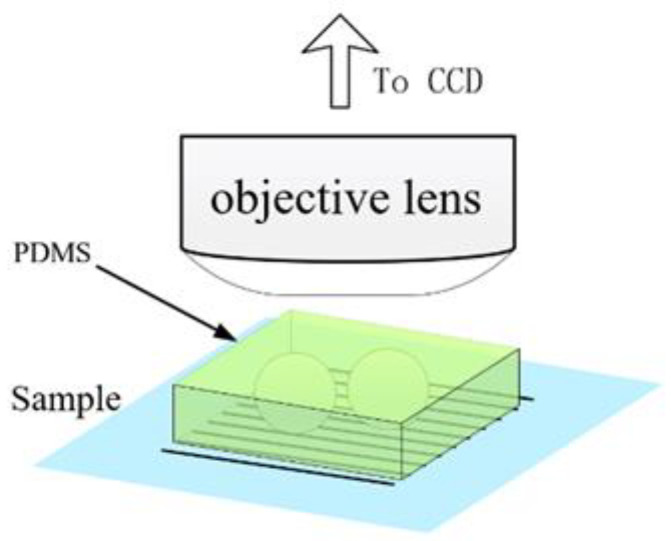
Imaging using PDMS thin-film encapsulated microspheres (PDMS thin-film encapsulated microspheres).

However, the volatility of the liquid used for immersion can lead to imaging instability, which limits prolonged observation of the sample. To cope with such instability in liquid immersion methods, Xiang Hao et al. proposed that the evaporation of liquid around the microspheres could be eliminated by increasing the hydrophilicity of the microspheres [24]. However, increasing the hydrophilicity of the microspheres requires treatment of the microspheres and adds volatilization uncertainty to the process, possibly affecting the image quality. Therefore, replacing the liquid layer with a non-liquid medium that is not volatile is a good solution. In 2014, Kenneth Allen et al. [25,26,27,28] used polydimethylsiloxane (PDMS) and high-refractive-index BaTiO_3_ microspheres to fabricate an imaging film with a correlation study. In 2015, Pang Hui [29] and a team at the Institute of Optoelectronics, Chinese Academy of Sciences, along with Arash Darafsheh et al. [30], adopted the idea and confirmed its feasibility using a film consisting of BaTiO_3_ microspheres and a PDMS soft film. PDMS, a flexible material formed after heat curing, is used as a transparent dielectric layer and eliminates the negative effects on imaging due to dielectric evaporation. At the same time, the closed nature of the PDMS film enables the large-area use of an encapsulated microsphere film. The diagram of the PDMS film-encapsulated microsphere method is shown in Figure 9.

Using this film to perform the corresponding super-resolution imaging experiments, Pang Hui’s team analyzed the effect of the refractive index of the media layer on microsphere imaging. They chose barium carbonate (BaTiO_3_ sphere) and silica microspheres (SiO_2_ sphere), with results showing that when the refractive index (n) of the media was between 1.33 and 1.548, silica microspheres only achieved resolution of sample features smaller than the diffraction limit at half submergence. The larger BaTiO_3_ microspheres needed to be fully submerged. The final imaging magnifications obtained experimentally are shown in Table 2.

The table shows that when the microsphere diameter is known, an appropriate reduction in the refractive index of the media layer obtains a larger imaging magnification. In summary, the main function of this imaging film is to provide initial magnification of samples smaller than the diffraction limit, allowing the samples to be resolved by the microscope. Therefore, a larger imaging magnification is favorable for observing smaller samples.

Due to the different imaging effects that can be produced when utilizing microspheres of different materials in combination with different media, a growing number of researchers are focusing their attention on the study of media microspheres. For example, in 2015, Yonghong Ye et al. [31] used experiments to demonstrate that sharper images can be obtained when microspheres are half-submerged in the medium. On that basis, in 2022, Jianguo Wang et al. [20] from Nanjing Normal University partially immersed PS microspheres in water, spin-on-glass, SU-8 resist, and S1805 resist. A comparison of the experimental results revealed that the highest resolution of the microspheres was achieved in the semi-immersed state, i.e., w½ 1/2 of the diameter of the PS microspheres was immersed in the medium. In the comparison of the experiments using microspheres immersed in different media, the PS microspheres half-immersed in SU-8 photoresist showed the least image distortion.

To clarify why immersed microspheres can achieve higher resolution, many researchers have analyzed them from a theoretical point of view.

For instance, in 2013, Yonghong Ye et al. [32] conducted a relevant research analysis on the far-field imaging capabilities of immersed microspheres. Subsequently, through experiments, they found that microspheres in a semi-immersed state capture more detailed information from the sample. Their research results contributed to an improved understanding of the microsphere imaging mechanism [33]. Later, in 2023, Jinzhong Ling et al. [34] at the Xi’an University of Electronic Science and Technology experimentally investigated microsphere imaging conditions, which is instructive for designing and optimizing microspheres for conventional optical microscopes using different immersion methods.

Many other studies exist, and researchers are still exploring and understanding other combinations of materials, media, and traditional microscopes to achieve better imaging results.

## 3. Microsphere Material and Preparation Process

### 3.1. Microsphere Materials

**Figure 10:**
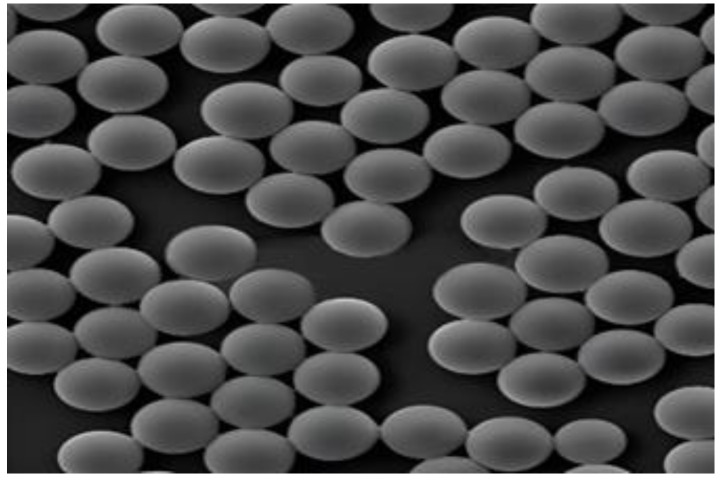
Typical silica microspheres.

Spherical objects are the most stable forms of matter that exist in nature. Microspheres are spherical or nearly spherical structural materials with diameters measured in nanometers and micrometers. Microspheres used for super-resolution imaging have smaller particle sizes and are generally made artificially. Typical microspheres are shown in Figure 10.

Microspheres are often made of silica, a desirable material due to its non-toxicity, odorlessness, non-fixed morphology, good optical transparency, chemical inertness, and biocompatibility. In addition to silica, barium titanate (BTG) and PS are also used by researchers. Table 3 shows some of the properties of microspheres made of these three materials. The photonic nanojet effect of microspheres is the basis of their imaging capabilities, and better imaging results are obtainable using microspheres with higher refractive indices. Microsphere imaging can be used to see not only the external features of a sample’s surface but also to detect changes in its internal physical properties. The latter finding extends the applicability of microsphere imaging to biomedicine.

### 3.2. Microsphere Preparation Process

Currently, the preparation technology of nanoscale microspheres is relatively mature, using pulverization, templates, precipitation, hydrothermal, droplet, and sol–gel methods. Hydrothermal, template, and sol–gel methods are the most common. For example, in 2003, Jiliang Yin et al. [35] prepared PS/TiO_2_ microspheres at 105 °C using the hydrothermal method. The PS kernel was first prepared by emulsion polymerization and crystal seed polymerization. The PS microspheres were then submerged in an ethanol solution of tetrabutyl titanate, causing the amorphous TiO_2_ to coat the submicrometer-sized monodispersed PS microspheres. Different thicknesses of the coatings could be obtained by adjusting the concentrations of tetrabutyl titanate and PS, i.e., the microspheres could be calcinated to obtain different thicknesses of the hollow TiO_2_ shells according to the experimental needs.

**Figure 11:**
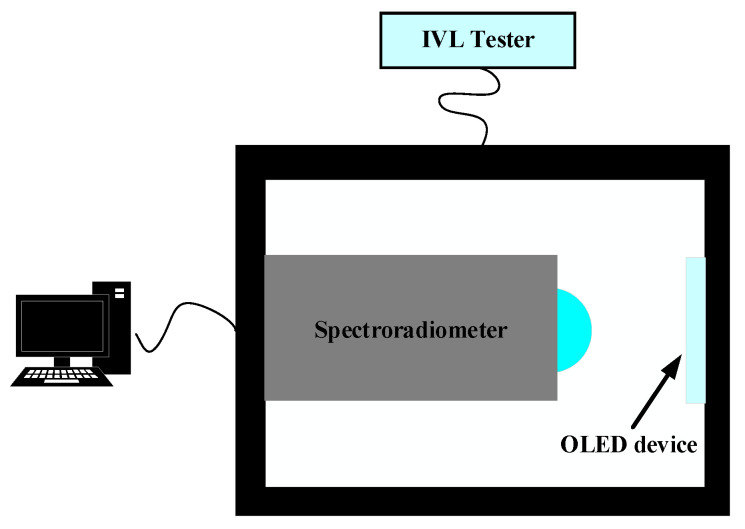
Measurement system and equipment used by Kim [36].

In 2013, Kim et al. [36] proposed the preparation of OLED microsphere lenses using microsphere templates, using the measurement system and equipment shown in Figure 11. The team used a PS colloid to make a single-layer mold, which showed a tightly distributed hexagonal stacking structure. After which, a PMMA mixture was poured into the mold, producing microsphere lens panels with diameters of 45–90 μm. The templates proposed in that study have great application potential.

When using the sol–gel method to prepare microspheres, an alcoholic or inorganic salt of the metal is first hydrolyzed to form a solution. The solute collection is then gelled and subsequently made into a film or dried directly. Finally, it is heat-treated to remove the organic components therein to obtain the microspheres required for the experiment.

Table 4 presents the advantages and disadvantages of the three common methods used for microsphere preparation.

## 4. Microsphere Manipulation

**Figure 12:**
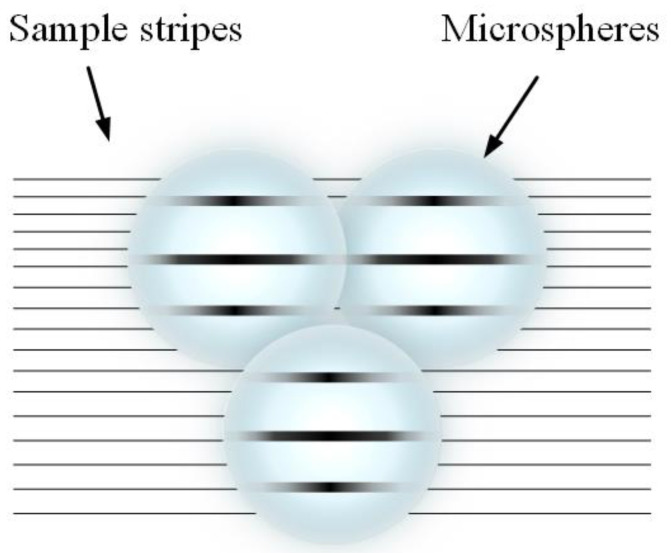
Microsphere array.

When using microspheres for imaging, individual microspheres have a limited imaging field of view. To view the sample from all sides and obtain images at different angles, single or multiple microspheres need to be moved and manipulated to place them in different angular positions for imaging. Microsphere manipulation technology arranges the microspheres in the desired order, with the spheres close together but not overlapping, and each sphere can collect information about the sample located below it. This information is then received by the microscope located above. Thus, when a plurality of microspheres completes an array-type close arrangement as described earlier, it is possible to image a large area of the sample located below the microspheres using an array, as shown in Figure 12. This design implements effective control of the microspheres, enhances their utility, and enables super-resolution imaging of an arbitrary area of the sample while expanding the imaging field of view. Past methods of randomly scattering microspheres for imaging samples have gradually been phased out, supplanted by various microsphere manipulation methods.

Existing microsphere manipulation techniques mainly include mechanical, chemical, and optical.

### 4.1. Mechanical Control Methods

**Figure 13:**
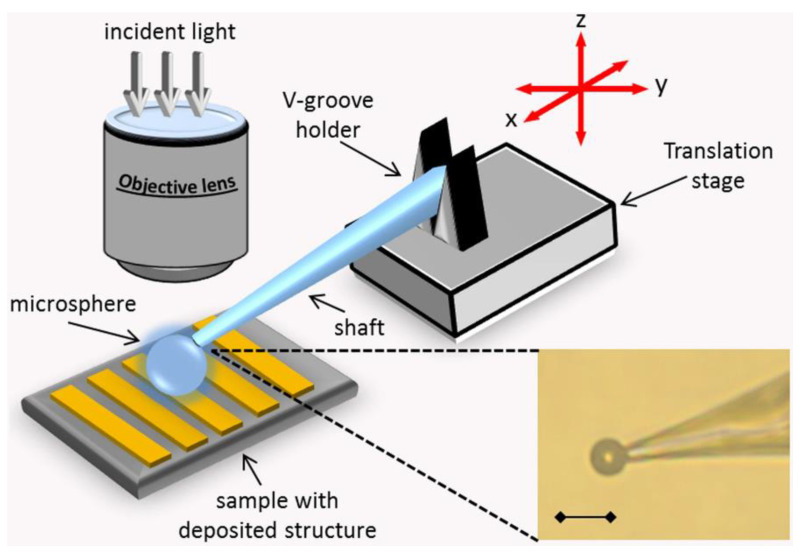
Structure of a capillary microprobe-driven microsphere experiment [38].

In 2013, Shuying Wang et al. [37] from Zhejiang University used capillary microprobes to manipulate microspheres. In the same year, Zengbo Wang collaborated with Leonid A. Krivitsky et al. [38] to enable capillary-driven microspheres for imaging arbitrary regions. A diagram of microsphere manipulation is shown in Figure 13, enabling any region of the observed object to be imaged, which is a qualitative improvement to the practical use of microspheres. This method manipulates microspheres by immobilizing them at the tip of a capillary microprobe. The tip of the capillary microprobe is only a few hundred nanometers or so and does not affect the optical path changes of the microspheres themselves. The advantage of the capillary microprobe over the traditional direct seeding method is the ability to move the microspheres away from the surface of the sample without touching or contaminating the sample.

**Figure 14:**
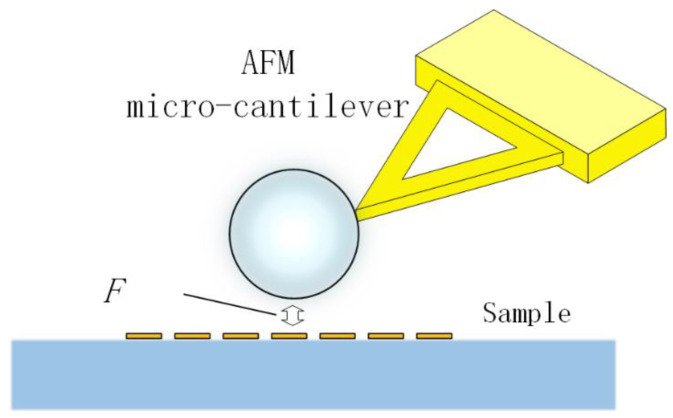
AFM microcantilever-driven microspheres.

However, the fine size of the capillary microprobe tip makes it difficult to perfectly control the distance between the microspheres and samples with rough surfaces. When the microspheres come into contact with the sample for imaging, the capillary may break at the tip due to uneven forces, causing the microspheres to detach. To address the drawbacks of capillary-controlled microspheres, Shuying Wang et al. again proposed a method using atomic force microscope (AFM) microcantilevers with microspheres [39]. In this method, microspheres are glued and fixed to the outer end of the AFM microcantilever, as shown in Figure 14. When microspheres approach the sample, they are constrained by forces such as Van der Waals forces within the range of atomic forces. This team successfully used this principle to arrange microspheres on their sample. The elasticity of the microcantilever allows the microspheres to follow the surface of the sample during contact imaging, ensuring that the microspheres are in a state of “soft contact”, effectively avoiding abnormal shedding of the microspheres from the microcantilever. The high sensitivity of the microcantilever also makes it suitable for imaging any area of the sample.

In summary, whether it is through a capillary microprobe or an AFM microcantilever, the essence of mechanical manipulation is to position the microsphere using the microprobe, thus ensuring precise placement while minimizing the risk of sample contamination.

Other technical issues with mechanical control remain. When moving the microspheres, unpredictable deviations in tool materials and operations coupled with the large number of components and the relatively large number of constituent components of the mechanical equipment present complex operational procedures and similar difficulties.

### 4.2. Chemical Manipulation Methods

**Figure 15:**
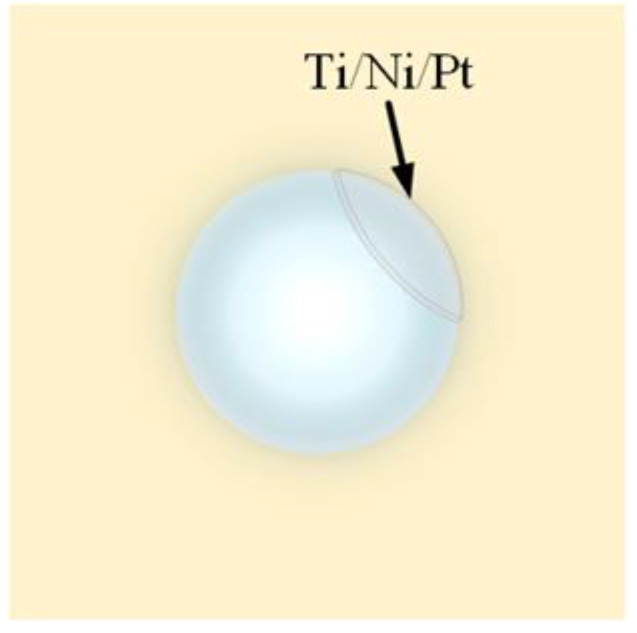
A microsphere-coated metal layer.

In 2016, Li and his team [40] developed a technique called “Swimming Microrobot Optical Nanoscopy”. This technique coats a Ti/Ni/Pt metal layer on the surface of a portion of the microsphere, as shown in Figure 15.

**Figure 16:**
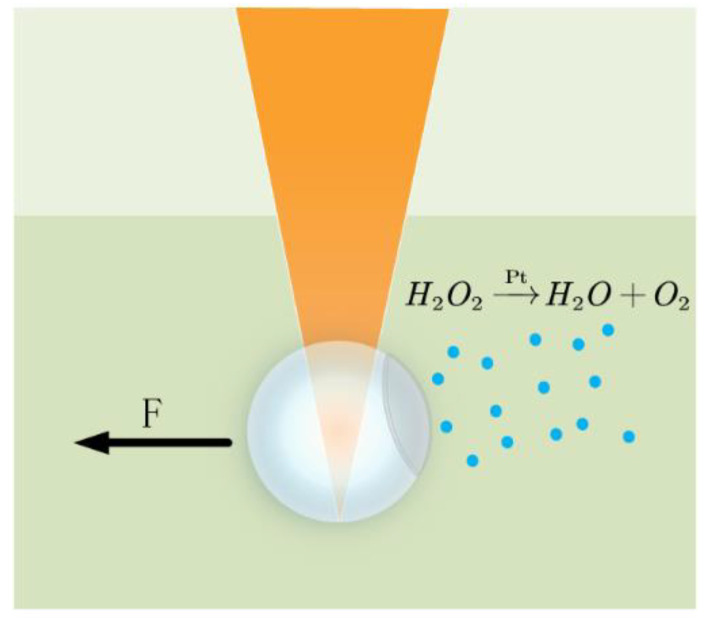
Chemically-driven manipulation of microspheres.

The treated microspheres were placed in a hydrogen peroxide solution, and Pt in the metal layer, a metal with a catalytic effect, was able to induce the decomposition of the hydrogen peroxide solution into water and oxygen. The resulting oxygen brought about a pressure difference that pushed the microspheres to move in the solution, as shown in Figure 16. At the same time, metal Ni is magnetic and can be attracted by external magnets, indirectly manipulating and driving the movement of microspheres. Using chemical local catalytic reactions to drive and magnetic forces to guide the movement of microspheres on the surface of the sample, it is possible to scan a large area. This autonomous microsphere technology is an efficient and easy-to-use nano-imaging technology.

However, the chemically driven method requires coating the surface of the microsphere with a metal, limiting the imaging field of view, and possibly altering the physical properties (e.g., the refractive index) of the microsphere itself and reducing the image quality.

### 4.3. Optical Manipulation

**Figure 17:**
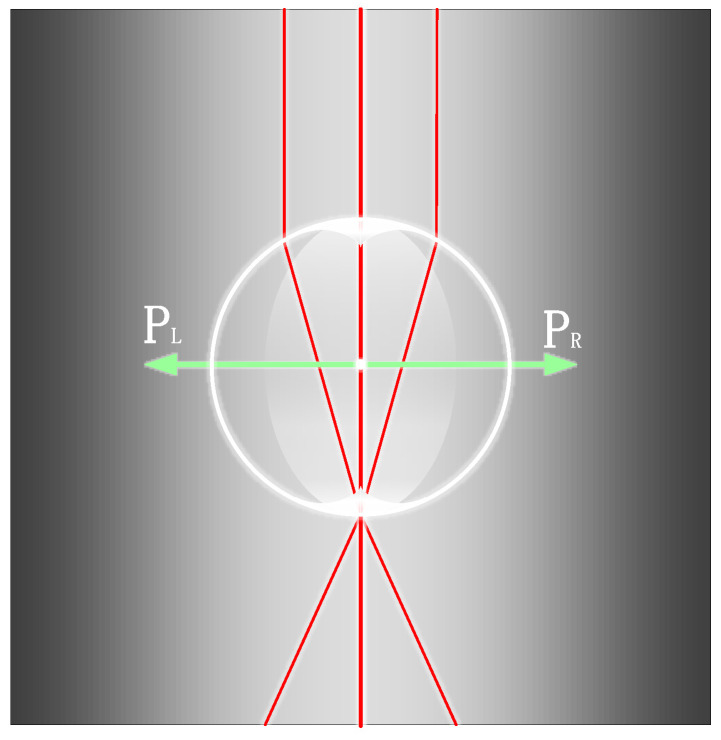
Forces on the microsphere in a light beam.

**Figure 18:**
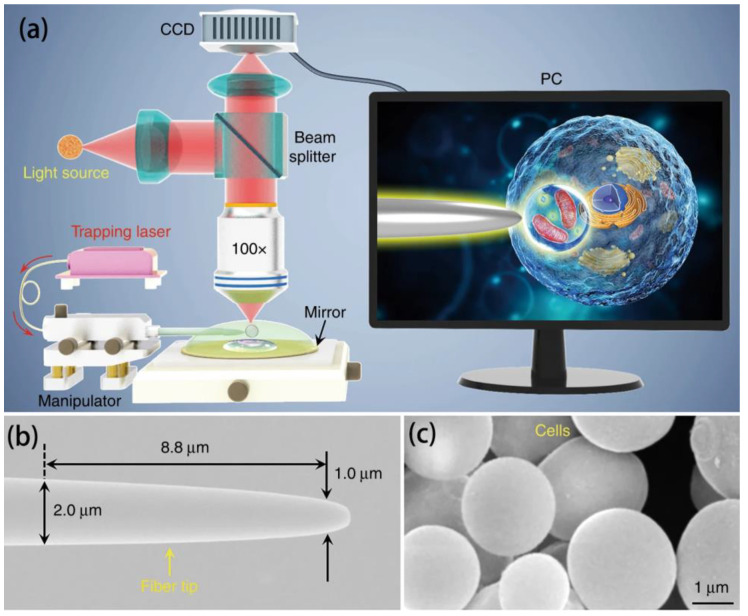
(**a**) Schematic of the experimental setup. (**b**) SEM image of a fiber optic tip with a tapered end diameter of 1.0 µm. (**c**) SEM image of a yeast cell-based biomagnifier with a smooth surface and spherical shape [41].

Unlike mechanical and chemical manipulation, optical manipulation is a contactless microparticle manipulation technique, typically represented by optical tweezers. In optical tweezer technology, the captured microparticles need to have a higher refractive index than the surrounding medium, which is typically the case with microsphere imaging. Therefore, optical tweezer technology has received special attention. Lasers are highly directional, monochromatic, and highly luminous, providing an ideal foundation for optical tweezer technology. Under powerful laser irradiation, particles can be trapped in an optical potential well, as shown in Figure 17. By regulating the nature of the light field, the result of the interaction between light and matter will change. This is the theoretical basis of optical tweezer technology. Using this purely optical manipulation technique, there is no contact with the microspheres at all. For example, in 2019, Yuchao Li et al. used fiber-optic tweezer technology to use captured cells as a biomagnifier, and the optical potential well formed by the focal point of this biomagnifier allowed for the precise manipulation of individual nanoscale particles with a radius of 50 nm. Its use of cells enables the manipulation of microspheres with a resulting optical resolution of 100 nm. The experimental setup and material characterization are shown in Figure 18 [41].

However, there are still problems with optical manipulation technology that need to be addressed. First, microspheres, as the core devices for optical tweezer manipulation and microsphere super-resolution imaging, have properties such as a refractive index that affect both optical trapping efficiency and imaging performance. Therefore, an in-depth analysis of the effects of the microsphere’s properties on these two factors to balance the capture performance with image resolution remains a major challenge for researchers to address. Second, existing optical tweezer technology often involves manipulating independent individual microspheres, limiting an inflexible imaging field of view, and resulting in low imaging efficiency. Although the field of view can be increased by increasing the microsphere size, there is a consequent decrease in resolution in that case. In addition to this method, whether the optical potential well can simultaneously manipulate multiple microspheres for imaging large areas of a sample is still a question that needs to be studied. Finally, high-refractive-index BTG microspheres increase the difficulty of non-contact manipulation compared with low-refractive-index microspheres, such as SiO_2_ and PS, resulting in microspheres that are difficult to capture using optical tweezers. Thus, improving the efficiency of optically manipulating BTG microspheres while maintaining their imaging advantages is still a difficulty.

Table 5 summarizes the advantages and disadvantages of the three manipulation types.

## 5. Potential Application Areas

**Figure 19:**
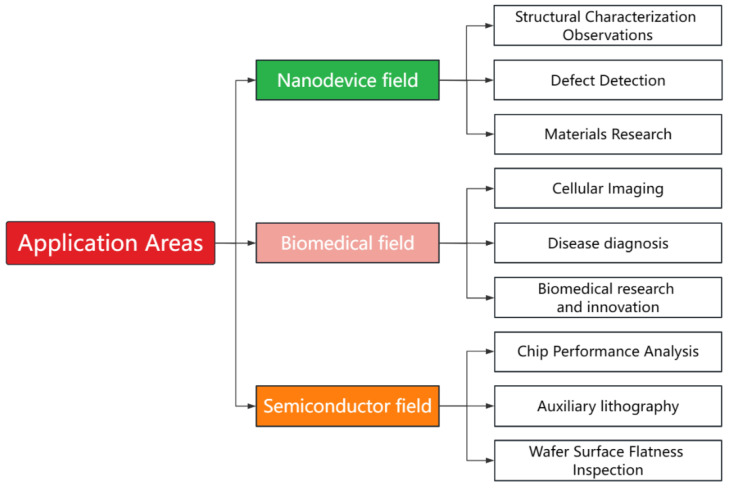
Potential applications of microsphere imaging.

Although microsphere imaging technology is still immature, continued research efforts are expected to discover multiple roles for the technology in multiple fields with significant commercialization potential. Potential application areas include nanodevices, biomedicine, and semiconductors, as shown in Figure 19.

### 5.1. Nanodevices

Nanodevices refer to devices with dimensions measured in nanometers. In this field, microsphere imaging is very important, particularly in the following aspects.

**Figure 20:**
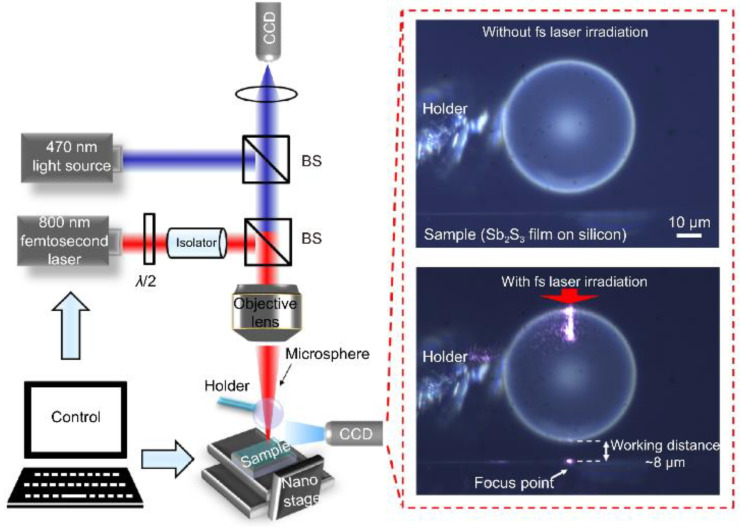
Experimental setup and side view of a femtosecond laser beam focused on microspheres [44].

1. Observation of structural characteristics: The small size of components of nanoscale structures makes observation using traditional methods difficult. Microsphere imaging can help researchers observe and analyze small devices’ appearance, morphology, and size. For example, in 2021, Liu et al. [42] used microsphere-assisted super-resolution imaging technology to perform super-resolution imaging on multi-layer samples, proving that it is capable of fault scanning in the imaging of multi-layer structured samples. The same discovery was made in 2023, when Hong Minghui et al. [43] utilized the superior resolution and label-free properties of optical microsphere nano mirrors to uncover their enormous potential in detecting sample morphologies.
2. Defect detection: The manufacture of nanodevices often has unavoidable small defects that are not visible using traditional imaging. Microsphere imaging enables better observation of the details of such tiny devices, with better detection and localization of these defects at the nanometer level. This can help manufacturers identify and resolve potential defects promptly to improve product quality. When using microsphere-assisted super-resolution imaging technology to scan and image samples, researchers can obtain a comprehensive view of the sample, thereby better realizing defect detection [42].
3. Materials research: Microsphere imaging can be used to analyze nanomaterials. Nanomaterials have special properties such as quantum effects. Using microspheres, the interaction between nanomaterials and the surrounding environment can be observed and explored to promote the development of nanotechnology. As an example of contemporary research, academician Minghui Hong and the team of Dun Cao [44] jointly reported an ultrafast laser nanopatterning technique using non-contact microspheres in 2023. The experimental setup with a side view of a femtosecond laser beam focusing on the microspheres is shown in Figure 20. The team prepared nanogratings that demonstrated ideal beam diffraction properties. This nanoscale resolution has significant implications for next-generation laser nanolithography in both far field and ambient air.

In summary, microsphere imaging has an important role to play in promoting the research, fabrication, and application of nanodevices.

### 5.2. Biomedicine

In the field of biomedicine, microsphere imaging is a very important and useful tool in the following areas.

**Figure 21:**
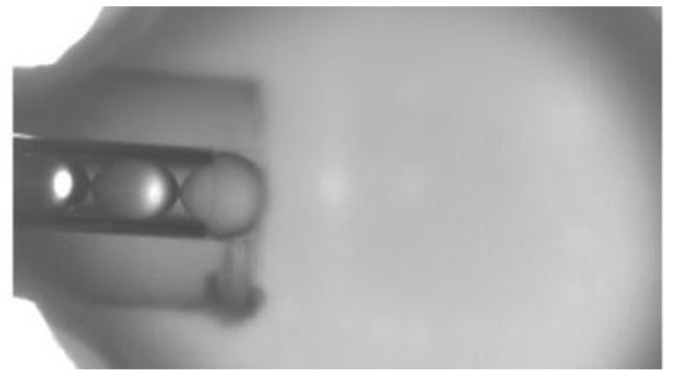
Microprobe insertion into the gel medium [45].

1. Cellular imaging: Microsphere imaging is capable of discriminating fine tissue structures, and researchers can use this ability to conduct in-depth studies on the structure and function of cells. For example, in 2010, Arash Darafsheh et al. [45] used a gel-like medium to simulate the optical properties of biological tissues, as shown in Figure 21.

**Figure 22:**
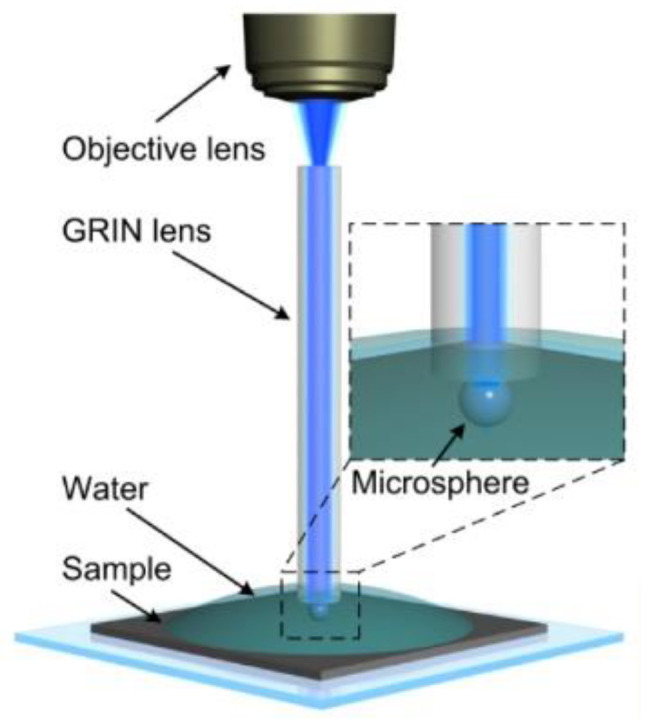
Experimental structure of a microsphere-based super-resolution endoscope [48].

In 2013, Lin Li et al. performed imaging of an adenovirus by submerging microspheres [46]. In 2015, Arash Darafsheh et al. [47] at the University of Pennsylvania demonstrated that microsphere-assisted super-resolution microscopy is feasible for examining biological and photonic structures, with the team working to expand the use of microsphere imaging in cancer and medical sciences. The observation of deep tissues has traditionally been limited to the cellular level, but in vivo image details at the subcellular and even organelle levels have been of great interest. Feifei Wang et al. of the Chinese Academy of Sciences reported a new endoscopic approach [48] in 2023, using microsphere-functionalized gradient-refractive-index (GRIN) lenses in combination with endoscopes. The experimental structure, shown in Figure 22, used real-time white light or fluorescence imaging and increased the resolution of traditional endoscopes.

**Figure 23:**
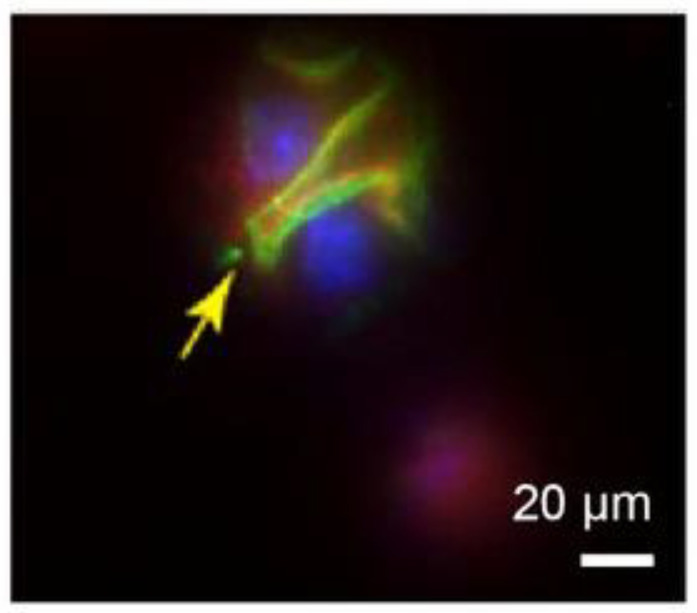
Glomerulus observed through microsphere focusing after staining [49].

**Figure 24:**
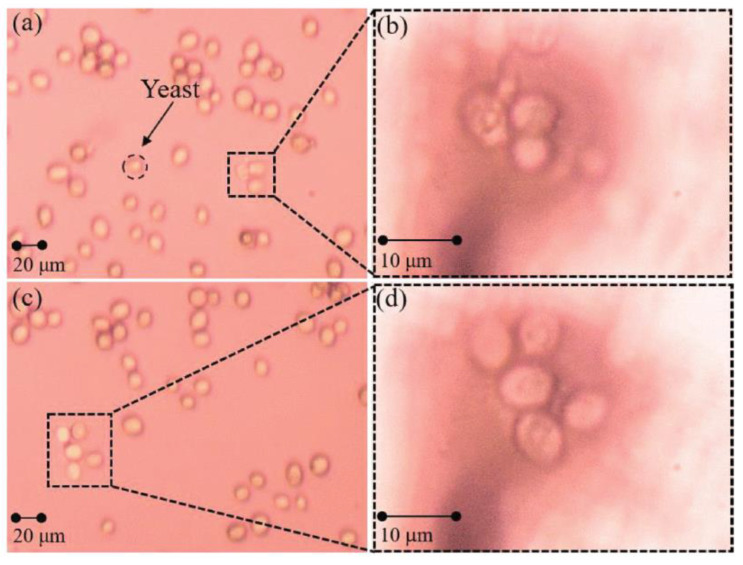
Images of free yeast cells in water. (**a**,**c**): Images captured by optical microscope. (**b**,**d**): Images captured by microspheres [50].

2. Disease diagnosis: High-resolution imaging of tissues and organs by microsphere imaging can provide more accurate and detailed information helpful for the diagnosis and assessment of diseases along with the study of physiological and pathological processes of tissues and organs. For example, in 2015, Arash Darafsheh et al. [49] developed a biological super-resolution imaging method using microspheres embedded in cover slips. Immunostaining was performed on renal slices, and significant enhancement of protein distribution patterns was observed in the stained glomeruli using microsphere-assisted super-resolution imaging technology. The stained glomeruli are shown in Figure 23. This method can be used to observe visceral details and diagnose diseases accordingly.

3. Biomedical research and innovation: Microsphere imaging technology goes beyond the limits of traditional imaging, improving the contrast and clarity of images and producing higher-resolution output. These improvements help researchers to understand biological mechanisms more fully and to promote the development and innovation in the field of biomedicine. For example, in 2019, Gao Shilin et al. [50] reported that, in addition to biological sectioning, probe-bound microspheres can also be imaged on free cells in water. Using a yeast system as the experimental object, the enlarged results are shown in Figure 24.

### 5.3. Semiconductors

**Figure 25:**
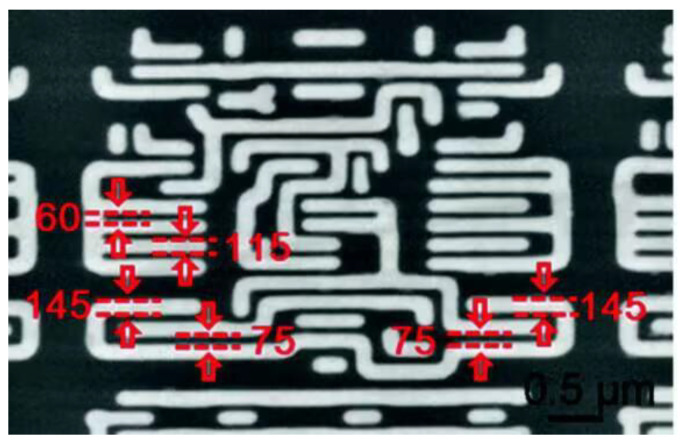
SEM image of a semiconductor chip [51].

In 2015, Haie Zhu et al. achieved a resolution of 60 nm using a wide-field imaging mode and 50 nm in a confocal mode for semiconductor chip samples using microsphere imaging. This far exceeded the resolution limit of conventional optical microscopy [51]. The chips used for the experiments have nanopatterned structures with 60 nm and 75 nm gaps and widths of 115 nm and 145 nm, as shown in Figure 25.

The small sizes involved in semiconductor manufacturing mean that microsphere imaging has many applications in the field as follows.

1. Chip performance analysis: Microsphere imaging can be used to probe the emission and absorption properties of photons in semiconductor materials, and it can also be used to characterize, analyze, and even visualize chip structures, so as to obtain more accurate information about chip performance. In 2023, Hong Minghui et al. [43] utilized the superior resolution and label-free properties of optical microsphere nano mirrors to detect integrated circuit chips in the semiconductor industry and to detect morphological characterization in the field of biology. It was proven that a microscale composite lens configuration composed of ultra-half microspheres can achieve optical imaging below 3 nm in ambient air when coupled with an objective lens (NA = 10.0), thereby improving the imaging ability of traditional microscopes by about an order of magnitude.
2. Assisted lithography: Lithography is an important step in the semiconductor manufacturing process. The use of microsphere imaging can assist lithography to improve the performance of semiconductor devices.
3. Wafer surface flatness detection: In the chip manufacturing process, the rough surface of a cut wafer surface needs to be ground and polished in a series of processes before it can be used for subsequent operations. Microsphere imaging has a wide field of view and can effectively examine wafers. For example, Wu et al. [52] demonstrated the use of microsphere lithography technology to manufacture and characterize nanopillar arrays on GaN substrates in their work. This confirms the applicability of microsphere-assisted super-resolution imaging technology on wafers.

There are also distinct technical differences in using microspheres for super-resolution imaging across different application fields. In the field of nanodevices, technological differences are mainly reflected in imaging accuracy, as this field may involve imaging structures at the nanoscale or even sub-nanoscale with stringent precision requirement. In the field of biomedicine, technological differences mainly arise in sample preparation and imaging condition optimization, as biological samples usually require labeling, fixation, or staining to enhance imaging contrast. In addition, it is necessary to consider the sample activity and the biocompatibility of microsphere materials to avoid adverse effects on biological samples. In the field of semiconductors, technological differences are mainly reflected in imaging resolution. Semiconductor devices typically have complex structures and surface details, and therefore require exceptionally high imaging resolution to ensure reliable imaging results. In summary, technological differences in applications across different fields are evident in their sample characteristics and imaging requirements. Despite these technical differences, the basic principles and core technologies underlying microsphere super-resolution imaging remain similar, as they all utilize the unique optical properties of microspheres to achieve super-resolution imaging. In conclusion, microsphere imaging technology has many potential applications in multiple industries and tremendous capabilities to offer researchers. Future developments in microsphere imaging are expected to develop new applications further still. With such developments, it is expected that the market demand for microspheres will continue to increase, with the microsphere industry becoming strategically important over time.

## 6. Research Trends

Research trends in microsphere imaging technology fall into several categories as follows.

1. Understanding the mechanism of microsphere imaging

Although some innovative research results have been achieved in microsphere-based imaging, most of them are still in the laboratory research stage. Existing studies have shown that factors such as the immersion method, materials, and process parameters have a great influence on imaging performance. However, the lack of complete and systematic theoretical support leaves our understanding of the principles insufficient for quantitative analysis and large-scale application of the technology.

2. Development of more microsphere materials and optimization of preparation processes

Existing studies have shown that surface smoothness, morphology, particle size, homogeneity, refractive index, and other microsphere parameters have a great influence on the imaging quality, so the development of more microsphere materials will be a focus of future development. In addition, different materials combined with different immersion methods also present completely different imaging qualities. For example, silica microsphere lenses achieve higher imaging resolution when semi-immersed in alcohol, and BaTiO_3_ microsphere lenses improve their resolution when fully immersed in oil. Therefore, the development of more microsphere materials and combinations with immersion liquids and methods deserves further investigation.

Meanwhile, existing microsphere preparation methods have a complicated, lengthy, and costly preparation process. Therefore, researchers need to find ways to solve these problems.

3. Building new types of control methods

To extend the field of view of microsphere imaging, it is necessary to manipulate the microsphere. However, existing mechanical manipulation methods are relatively complex and error-prone, chemical manipulation methods easily contaminate samples and reduce the image quality, and optical manipulation methods limit the field of view. Developing a new type of manipulation that is cheaper, simpler, and cleaner is worth further exploration and research. Preliminary research shows that magnetic control is flexible, harmless, and convenient without the need to coat the microspheres. It has been used in the fields of micro-robotics and particle manipulation, but it has not been applied in the field of microsphere manipulation thus far. Further research into this possibility for microsphere imaging is warranted.

4. Expanding areas of application

Although microsphere-based imaging technology is still in the early exploration and laboratory research and development stages, it has already demonstrated tremendous potential in the fields of nanodevices, biomedicine, and semiconductors. It is not difficult to predict its further expansion with the developments in nanotechnology, reaching into areas such as microfluidics, environmental monitoring, data storage, and other fields, as key challenges are solved.

5. Addressing the limitations of microsphere-based super-resolution imaging systems

In the future, several directions can be explored to overcome the limitations of imaging systems. These include developing specially designed optical systems to maximize the utilization of the super-resolution characteristics of microspheres for imaging, better realizing sample positioning techniques to ensure the precise positioning of microspheres, designing anti-scattering microspheres to reduce light distortion and improve super-resolution imaging results, and advancing microsphere preparation methods to reduce costs. We believe that continuous research and innovation can accelerate the development of super-resolution imaging technology.

## 7. Conclusions

Microsphere super-resolution imaging techniques break through the diffraction limits of traditional microscopy with the advantages of low cost, simple operation, and high resolution. However, the technology is still immature, and further research to understand the underlying principles is still needed. Improving our understanding is expected to be beneficial for combining microspheres and their immersion methods to obtain better imaging results. In addition, the optical properties of the microspheres themselves have greatly influenced the results, so the preparation of microspheres should not be underestimated. When using a single microsphere to observe a sample, the field of view is often limited, making it necessary to manipulate the microspheres into arrays to expand the field of view. However, it is important to avoid contaminating or damaging the sample with microspheres. This is leading to a growing interest in the practical application of the microsphere manipulation method. In any case, the manipulation of microspheres is a key research priority. In conclusion, the gradual maturation of microsphere super-resolution imaging technology is predicted to offer many innovative and important uses in fields such as biomedicine, nanodevices, and semiconductors in the future.

## Figures and Tables

**Table 1:** Comparison of super-resolution imaging techniques.

Super-Resolution Imaging	Imaging Speed	Sample Processing Requirements	Field of Application
STED	Slow	High	Samples are often labeled and fixed for use in areas such as biomedical and materials science.
SIM	Fast	Low	Samples are typically less demanding to prepare; suitable for cell biology and neuroscience.
SMLM	Slow	High	Often requires special labeling and handling of the sample; suitable for fields such as single molecule level localization tracking.
Microspheres	Fast	Low	Suitable for all types of samples, including fixed samples and biological cells.

**Table 2:** Imaging magnifications obtained from experimental tests [29].

Sphere	*n* = 1.33	*n* = 1.38	*n* = 1.548
SiO_2_	1.8×	1.67×	1.44×
BaTiO_3_	3.96×	2.88×	2.11×

**Table 3:** Comparison of the performance of three common microspheres.

Microsphere Types	Transmittance	Index of Refraction	Chemical Inertness	Biocompatibility
Silicon dioxide microspheres (SiO_2_)	Highest	Lowest	Best	Good
Barium titanate microspheres (BaTiO_3_)	Medium	Highest	Bad	Medium
Polystyrene Microspheres ((C_8_H_8_)_n_)	High	Low	Good	Bad

**Table 4:** Comparison of microsphere preparation methods.

Preparation Method	Advantages	Disadvantages
Hydrothermal method	Simple operation, controllable structure, morphology, and purity of the prepared microspheres.	The preparation process requires high temperature and high pressure, and many organic compounds are required as reaction materials, resulting in high cost and energy consumption. The purity and uniformity of microspheres will be affected.
Template method	The preparation process is simple, and the morphology and size of the prepared microspheres are controllable, with good performance. Large molds can be used for large-scale preparation.	When preparing microspheres, it is necessary to prepare the mold in advance. If different sizes of microspheres are needed, a separate mold needs to be prepared, which is not easy to adjust, and the disinfection and cleaning of the mold are more complicated.
Sol–gel method	Easy to operate, fewer side effects, and easy to control the process.	During the preparation process, microspheres are prone to aggregation and adhesion, and the monodispersity of the prepared microspheres is poor, requiring a longer preparation time.

**Table 5:** Comparison of microsphere manipulation methods.

Method	Advantages	Disadvantages
Mechanical	The microspheres can be precisely maneuvered to the target position above the sample to be observed, flexibly maneuvered to move during observation, and flexibly withdrawn at the end of the observation to avoid contamination of the sample.	These methods have a relatively large number of components and complex processes that result in unpredictable deviations in tool materials and operations.
Chemical	It is possible to move the microspheres to the destination location from a distance without contact using a chemical reaction.	The metal layer on the microsphere required by the method reduces image quality.
Optical	It is possible to control the movement of microspheres to their destination position without contact and destruction using optical tweezers technology.	Microsphere characteristics such as refractive index affect both optical capture efficiency and imaging performance. Additional problems include a limited imaging field of view, a high cost of equipment, and a complicated optical path design in optical tweezers technology.

## Data Availability

The data underlying the results presented in this manuscript are not publicly available at this time but may be obtained from the authors upon reasonable request.
